# Late breeding season definitive prebasic molt in males, and late breeding season brood care by females, in central California Wilson’s warblers

**DOI:** 10.1002/ece3.8689

**Published:** 2022-03-18

**Authors:** William M. Gilbert

**Affiliations:** ^1^ 17002 Department of Science and Mathematics Chabot College Hayward California USA

**Keywords:** basic plumage, *Cardellina pusilla*, molt migration, molting grounds, post‐molt singing, uniparental brood care, worn plumage

## Abstract

I made observations of a central California population of Wilson's Warbler, *Cardellina pusilla*, after July 1 over 10 breeding seasons. I sighted males in definitive prebasic molt from July 4 (in 2007) to September 1 (in 1999). Most territorial males molted on their breeding territories, and individual molt lasted up to 46 days. Following prebasic molt, territorial males engaged in subdued “post‐molt singing,” which lasted about 7 days in some males, and which I first heard on August 13 (in 2004) and last heard on September 6 (in 1999). I sighted no female in definitive prebasic molt, or in fresh basic plumage, during the study. Of 13 females sighted ≥ July 21, 11 were in late breeding season uniparental brood care, and I could not rule out late brood care for the other two. Most, and possibly all, females not engaged in late season uniparental brood care apparently vacated their breeding territories before July 21. This departure was much earlier than for resident males, the last of which I sighted on September 10 (in 1999). Early‐departing females presumably underwent prebasic molt after July 21 at locations not known. Remaining late‐nesting females must have molted much later than resident males and likely later than early‐departing females, and at locations unknown. I last sighted two uniparental brood‐tending females, still in worn plumage, on August 26 and 29, respectively. Two unique findings of this study are a male/female difference in location of prebasic molt, and a likely dichotomy of prebasic molt timing between females leaving their breeding territories early and those remaining in uniparental brood care. Another finding, post‐molt singing in most and possible all territorial males, is a largely unrecognized behavior, but one previously reported in several passerine species. Post‐molt singing may reliably indicate completion of prebasic molt.

## Graphical abstract

Field observation of late breeding season behavior of Wilson's Warblers, *Cardellina pusilla*, in central California, U.S.A., showed that resident males underwent definitive prebasic molt on their breeding territories, while resident females did not. Most resident females left the study area and apparently molted elsewhere, while some females remained on territory, uniparentally raised late season broods, but never were seen to begin prebasic molt. Most, and possibly all, territorial males engaged in brief post‐molt singing following completion of prebasic molt.

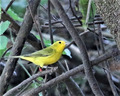

## 1 INTRODUCTION

There exists wide variation in the definitive prebasic molting regimens of migratory passerines. Many migratory passerine species undergo complete definitive prebasic molt, or at least the molt of flight feathers, on their breeding grounds before flying to wintering sites (Rohwer et al., 2005; Ryder & Rimmer, 2003; Vega Rivera et al., 1998). Other species undertake “molt‐migration,” which can involve a wide range of timing and locations (Flockhart, 2010; Pyle et al., 2018; Rohwer et al., 2008; Tonra & Reudink, 2018; Wiegardt et al., 2017), but often involves recognized “molting grounds” (Steele & McCormick, 1995; Tonra & Reudink, 2018). Finally, some passerine species migrate to wintering sites before beginning prebasic molt (Jenni & Winkler, 2020; Rohwer et al., 2005). Although these are considered to be the three basic strategies of definitive prebasic molt, at least in western North American passerines (Carlisle et al., 2005; Pyle, 1997), many variations occur, and altitudinal molt migration, in addition to latitudinal molt migration, is receiving increasing research attention (Pageau et al., 2020). Also, there can be wide interannual stochastic variations in molting strategies, including differences in timing and the proportion of individuals engaging in different strategies, and strategies can vary in response to environmental factors including breeding success and food supplies (Pageau et al., 2021).

Males and females of many passerine species initiate definitive prebasic molt at about the same time of season, and require about the same amount of time to complete molt, with differences varying by only a few days (Butler et al., 2008; Flockhart, 2010; Heise & Rimmer, 2000; Vega Rivera et al., 1998). In studies that have found significant sex difference in the mean timing of prebasic molt, most have found that males initiate and complete molt earlier than females (Borowske et al., 2017; Rimmer, 1988; Ryder & Rimmer, 2003; Svensson & Nilsson, 1997). However, few studies have reported mean migratory passage times, which potentially can reflect timing of definitive prebasic molt, to be earlier for females than for males in some species (Carlisle et al., 2005; Swanson et al., 1999). The greatest mean difference in timing of definitive prebasic molt between the sexes that I found in the literature, based on field observations, was a mean 12 days earlier for male Seaside Sparrows (*Ammospiza maritimus*, Borowske et al., 2017).

The majority of studies on definitive prebasic molting, or timing of autumn migratory passage which can relate to timing of molt, have involved late or post‐breeding season mist netting (Carlisle et al., 2005; Junda et al., 2020; Pyle et al., 2018; Ryder & Rimmer, 2003; Wiegardt, Wolfe, et al., 2017; Yong et al., 1998). One limitation of these studies is that they cannot directly evaluate the molting and behavior of individuals that possibly remain on breeding grounds after the majority of individuals have departed for migration. Thus, possible late nesting and delayed molting in those individuals cannot be assessed. Also, contrary to direct observation of a color‐banded and sexed study population, migrating, sexually monomorphic species can be difficult to sex by rapid, non‐invasive means in the autumn. Possibly based on that difficulty, and the fact that in many species timing of molt between sexes is minimal in any case, some mist‐netting studies have not distinguished molting data based on sex (Pyle et al., 2018; Wiegardt, Wolfe, et al., 2017).

**FIGURE 1:**
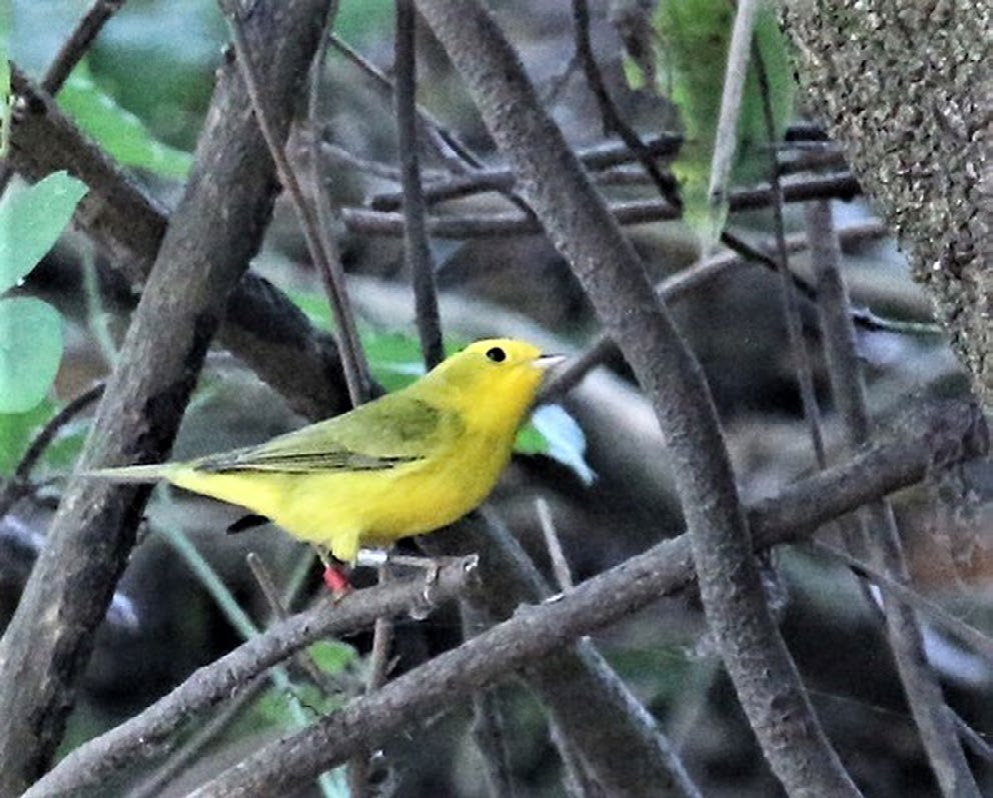
A color‐banded male Wilson's Warbler at my study site at the Tilden Nature Center in the East San Francisco Bay Area. Photograph taken in the wild by Erica Kawata

I here report observations and conclusions made during the late breeding season (>1 July) over 10 years of comprehensive field observation. This study was part of a broader study into the breeding ecology of Wilson's Warblers (Figure 1), carried out over a greater number of years. However, information here reported specifically relates to timing and behaviors associated with definitive prebasic molt and late breeding season nesting. Since this study was based on direct field observation, it has been able to reveal some facets of molting and late breeding behavior that studies mentioned above were not designed to explore, and some findings of this study vary from general consensus established by prior studies.

## 2 METHODOLOGY

### 2.1 Study site

My study site was approximately 0.18 x 0.26 km in dimension, and covered approximately 4.7 ha of parkland in the Tilden Nature Area, East Bay Regional Park District, Contra Costa County, California (38° N, 122° W.) The study site was located in an area of low hills and creek valleys to the east of San Francisco Bay, and was bordered on one side by Wildcat Creek, and on the uphill side by oak/bay woodland. The dryer upland woodland was populated mainly by Coast Live Oak (*Quercus agrifolia*) and California Bay Laurel (*Umbellularia californica*), and the riparian woodland along Wildcat creek was populated mainly by willows (*salix* sp.). Wilson's Warblers tended to prefer moist areas with at least some riparian habitat for nesting, but moved into dryer oak/bay woodland when population numbers were higher.

### 2.2 Timing and procedures

This study was part of a more extensive investigation of Wilson's Warbler breeding ecology extending from 1995 through 2017. For this study, I annually made observations, after July 1, of Wilson's Warbler breeding, molting, and plumages, over 10 study seasons, for a varying number of days among years (mean =#28.3±15.7 SD, range =#11–54) from 1998 through 2009 exclusive of years 2000 and 2005. In four field study years (1999, 2001, 2003, and 2004), I extended observations into September, with my latest observations on September 19 (in 1999). For identification of individuals, I captured Wilson's Warblers (mostly prior to July 1 every year; under BBL permit #22521) in 32 mm poly mist nets of 6 or 7 m length. I banded those Wilson's Warblers with a numbered F & W band on one leg and a unique combination of color bands, usually two, on the other leg. I captured most males in single mist nets, placed in their breeding territories, and accompanied by audio playback of recorded male song. I also captured many females in this way. I also captured some males and females in multiple nets placed in areas where individuals were active, often males chasing a nest building or exploring female. I determined sex, based on presence of cloacal protuberance or brood patch, for all after‐hatching‐year (AHY) captures, and determined second‐year or after‐second‐year age (SY or ASY respectively) for 32% (69/214) of AHY captures, based on condition of rectrices, presence or absence of molt limits in wing coverts (Pyle, 1997), and/or if a bird was a recapture from a previous year, in which case it was an ASY bird. However, in this study I did not use my aging data to evaluate behaviors. During the 10 years of my study, I obtained data from 40 color‐banded AHY females and 85 AHY color‐banded males (Table 1).

**TABLE 1:** Timing or tabulation of relevant information related to Wilson's Warbler late breeding season pre‐basic molt, plumage, or brood care

Relevant information	Dates or tabulations
Latest dates I sighted males in worn plumage (three samples)	21 July (in 1998, 2005), 22 July (in 1998)
Latest date I sighted a male in worn plumage and in biparental brood care	21 July (1998)
Earliest dates I sighted males in prebasic molt (two samples)	4 July (2007); 5 July (2008)
Latest dates I sighted males in prebasic molt (two samples)	26 Aug. (1999), 1 Sept. (1999)
Maximum number of days I sighted a given male in prebasic molt (two samples)	46 (#9924), 46 (#0112)
Extent, in days, over which I sighted males in prebasic molt	59 (4 July [2007] ‐ 1 Sept. [1999])
Total number of times I sighted males in prebasic molt over nine study seasons	35
Earliest dates I sighted and/or heard males in post‐molt singing (two samples)	13 Aug. (2004); 16 Aug. (2001, 2007)
Latest dates I sighted and/or heard males in post‐molt singing (two samples)	5 Sept. (2001), 6 Sept. (1999)
Latest dates I sighted silent banded resident males in mixed‐species foraging flocks (two samples)	6 Sept. (1999), 10 Sept. (1999)
Greatest length in days a male remained on territory after last heard in post‐molt singing	4 days (1999)
Extent, across years, over which I sighted males in basic plumage and/or heard them in post‐molt singing	28 days (13 Aug. [in 2004] ‐ 10 Sept. [in 1999])
Extent, across years, over which I heard males in post‐molt singing	25 days (13 Aug. [in 2004] ‐ 6 Sept. [in 1999])
Maximum extent, within a given year, over which I heard post‐molt singing	21 days (16 August ‐ 5 September 2001)
Total number of times I sighted males in fresh basic plumage and/or heard them in post molt singing	40
Greatest extent of days I sighted females in late‐season (on or after 21 July) fledgling care (two samples)	29 [21 July ‐ 19 Aug. [1999]), 36 (21 July ‐ 26 Aug. [1999]
Total number of different females that I sighted in the late breeding season (on or after 21 July)	13
Total number of different females that I sighted in worn plumage on or after 21 July	13
Total number of different females that I sighted in late season (on or after 21 July) uniparental brood care	11
Total number of times I sighted females in prebasic molt over nine study seasons	0
Total number of times I sighted females in fresh basic plumage over nine study seasons	0
Last dates I sighted females in worn plumage (two samples)	26 Aug. (1999), 29 Aug, (2003)
Earliest dates I observed Wilson's Warblerss to be totally absent from my study area (four samples)	3 Sept. (2003), 4 Sept. (2004), 7 Sept. (2001), 14 Sept. (1999)

This research followed a flexible procedural design involving an intent to comprehensively observe, record, and evaluate sighted behaviors on a daily basis. Field observation plans could change day to day, as I structured these plans based on an iterative process which considered what field observations might return the most important information based on prior observations.

### 2.3 Observations of male and female molt and plumage

I assessed the molt and plumage status of all AHY Wilson's Warblers sighted after July 1 during every observation year. I considered weak, fluttering flight, and/or hopping from twig to twig in vegetation less than 2 m height in most cases, to be indicative of molting flight feathers. I observed such compromised flight in multiple males from 1998 through 2009, based on observations lasting from about 30 sec to 18 min. Flight in these molting males was noticeably dissimilar from that seen in foraging individuals, which usually was rapid and darting. I also considered absence of rectrices to suggest, but not confirm, possible flight feather molt. Rectrices can be shed as a means of predator evasion (Awasthy, 2010; Møller et al., 2006). In this regard, I encountered three female Wilson's Warblers at my study site which lacked rectrices. I sexed the first as female through observing it construct two nests between June 10 and 19, 2001. The second, netted on June 21, 2006, I sexed as female based on its brood patch. The third, I sexed as female since it fed nestlings, along with its color‐banded male mate, between July 19 and 23, 2009. Other than their missing rectrices, none of the three females showed evidence of feather loss, or what Nolan (1978) called “noticeable molt.” Also, I observed no other females at my study site that displayed any feather loss, and two of the three tailless females I sighted had lost their rectrices before the date of first observed molting in any male (July 4, 2007). These observations confirmed that rectrices can be lost in Wilson's Warblers for reasons other than molting, and such loss thus could not be a reliable indicator of prebasic molt.

Detached contour feathers, often plucked loose by a preening male, I considered to be indicative of body feather molt. I also considered the contrast between worn, patchy body feathers and fresh basic plumage body feathers to indicate continuing prebasic body feather molt. All above‐mentioned conditions (weak, fluttering flight, shedding of body feathers, etc.) are indicative of noticeable molt (Nolan, 1978), observable in the field. I considered observation of either noticeable flight feather molt or contour feather molt, or both, to indicate that prebasic molt was in progress. Although I sighted a male beginning noticeable prebasic molt as early as July 4, a few males continued to provision nestlings past mid‐July while remaining in worn plumage, with no noticeable molt.

The contrast between males in noticeable molt and males that had completed prebasic molt was evident, and I considered both strong flight and fresh basic plumage to indicate that prebasic molt was complete or near completion. However, males in my study area also sang, usually in subdued volume, for short time periods after completing prebasic molt, and all such singing males that I sighted were in fresh basic plumage and flew well. I thus considered this “post‐molt singing” (Metcalf, 2003–2004) to be a reasonable indicator that prebasic molt was complete, even when an individual was not sighted.

In 1999 and 2001, on or after July 4, the first day I ever observed molting in males, I made concentrated efforts to observe molting in five and six color‐banded males, respectively, occupying territories centrally located in my study area. If I had not yet observed one of these targeted males in prebasic molt, or had not yet heard it in post‐molt singing, I spent additional time observing its territory, and made additional passes by the territory during observation days. The purpose of these more intensive searches was to learn the likely extent to which prebasic molt in males might happen on their breeding territories, as observing the extent of molting in these samples likely could be applied to males in the entire study population. In addition to information related to molt in males, I recorded the sighting dates, molt and plumage status, and any late season brood care of females sighted during the late breeding season on or after July 21.

### 2.4 Statistical analyses

I applied descriptive statistics to determine means and standard deviations of number of days I annually observed for molt‐ and plumage‐related behaviors during the 10 years of field observation. I used Fisher's exact test to evaluate the difference between the proportion of males remaining vs. not remaining on their breeding territories late into the breeding season, compared with the proportion present earlier, prior to prebasic molt. I also used Fisher's exact test to similarly evaluate the proportion of females remaining vs. not remaining on their breeding territories late into the breeding season, compared with the proportion originally present. Finally, I used Fisher's exact test to evaluate the difference between the frequency of males and females seen molting on their breeding territories.

## 3 RESULTS

### 3.1 Timing and magnitude of plumage and molt

**FIGURE 2:**
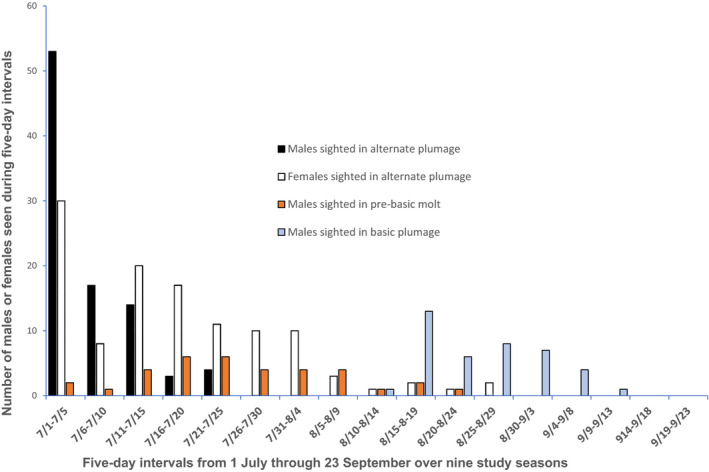
Cumulative number of sightings over ten study seasons of male and female Wilson's Warblers in different plumage or molting stages during five‐day intervals between 1 July and 23 September. I sighted no females molting or in fresh basic plumage

I illustrate the timing and magnitude of plumage and molting observations in Figure 2, and state the dates or numerical tabulations of relevant observations in Table 1. Over the 10 years of my study, I sighted 41% (35/85) of color‐banded territorial males at my study site to be molting and/or in fresh basic plumage (Table 1). I observed AHY males in prebasic molt multiple times during 8 of the 10 years of my study, and observed them in fresh basic plumage and/or in post‐molt singing multiple times during 6 of the 10 years. I observed definitive prebasic molt in a male on its breeding territory as early as July 4 (in 2007), and I did not observe a male in worn plumage later than July 22 (in 1998). All sighted territorial males initiated prebasic molt between early and late July (Table 1), and ceased or greatly reduced (one case) brood care after starting molt.

Of 40 color‐banded territorial females at my study site over the 10 years of my study, I sighted eight retaining their worn plumage into the late breeding season (≥ 21 July), and saw none in prebasic molt or fresh basic plumage in observations that lasted into September (Figure 2, Table 1). I additionally sighted 5 unbanded territorial females retaining their worn plumage for a total of 13 late breeding season females that had not undergone prebasic molt or attained fresh basic plumage. Based on the great difference in respective number of each sex that I sighted in prebasic molt and/or fresh basic plumage, there was a highly significant difference (*p* < .0001, Fisher's exact test, Table 2a) between the sexes in terms of late breeding season plumage and prebasic molt at my study site.

**TABLE 2:** (a) Fisher's Exact test analysis of the frequencies of prebasic molt and/or fresh basic plumage sighted in male vs. female Wilson's Warblers occupying breedng territories in the late breeding season (≥ 21 July). *P* < .0001 that frequencies are similar. (b) Fisher's Exact Test analysis of the frequencies of territorial occupancy by color‐banded male Wilson's Warblers during the early breeding season (< 21 July) vs. the late breeding season (≥ 21 July). *P* = 1.0000 that frequencies are similar. (c) Fisher's Exact Test analysis of the frequencies of territorial occupancy by color‐banded female Wilson's Warblers during the early breeding season (< 21 July) vs. the late breeding season (≥ 21 July). *P* < .0001 that frequencies are similar

(a)	Males sighted	Females sighted	Total
Molting	35	0	35
Not molting	0	13	13
Total	35	13	

(b)	Males sighted	Males not sighted	Total
Early season	11	0	11
Late season	10	1	11
Total	21	1	

(c)	Females sighted	Females not sighted	Total
Early season	40	0	40
Late season	8	32	40
Total	48	32	

### 3.2 Continued occupancy of breeding territories by male and female Wilson's Warblers

Of five males holding territories central to my study area in 1999, for which I searched intently following their last observed brood care, I sighted four at least once, and never sighted the fifth male. Of six males for which I similarly searched in 2001, I sighted all six at least once. Fisher's exact test (Table 2b) indicated no significant difference (*p* = 1.000) between the proportion of males sighted vs. not sighted on their breeding territories prior to July 4, compared with the proportion of males sighted vs. not sighted on their breeding territories on or after July 4. These results indicate that a significant proportion of territorial males remained on their breeding territories late into the breeding season, and after concluding their active breeding. Furthermore, observations indicated that those color‐banded males remaining at the study site were the majority, since 91% (10/11) of the centrally located sample of resident males remained on their breeding territories after July 4.

I sighted 13 females remaining on their breeding territories on or after July 21, of which 8 were color banded and 5 were unbanded. Fisher's exact test (Table 2c) indicated a significant difference (*p* = .0001) between the proportion of color‐banded females sighted vs. not sighted on their breeding territories prior to July 21 compared with the proportion sighted vs. not sighted on their breeding territories on or after July 21. These results indicated that a significant number of territorial females vacated their breeding territories after concluding active breeding. Furthermore, observations suggested that those color‐banded females which had left the study site before July 21 were the majority, since I sighted just 20% (8/40) of color‐banded females remaining at the study site on or after July 21. I confirmed six of those eight color‐banded females to be raising late breeding season uniparental broods.

### 3.3 Late breeding season brood care

I confirmed that 11 of 13 females remaining at the study site on or after July 21 were brooding eggs and/or caring for nestlings. Nine of the 13 females sighted on or after July 21 had been paired with territorial males, and they all had tended earlier biparental broods. For the four other females, I saw no evidence that they had been paired with territorial social mates. However, I had observed each of those four females uniparentally tend an earlier brood. Of the nine females which tended late broods and had been paired with territorial social mates, eight received no assistance from their mates in caring for their late breeding season broods. For one female, however, the social mate showed up during the last 2 days of brood care and, although molting and with compromised flight, it fed the brood 19.5% of the time (23/118 food deliveries) over about 2.5 hours of observation. Their brood was the only late breeding season brood to contain four young, while all others contained two or three young.

### 3.4 Breeding success of late‐nesting uniparental females

Of the 11 females confirmed to have been involved in late breeding season (≥ July 21) brood care, 9 fledged their broods, and 2 nests were depredated. This late breeding season nesting success rate (82%, 9/11, from 1998 to 2009) by primarily uniparental females was higher than nest success rates determined for early breeding season biparental broods at my study site (16 %, from 1995 to 1998; 58% in 1999; Ammon & Gilbert, 1999). Most broods tended in the late breeding season by primarily uniparental female nesters were of reduced size (mean = 2.83±0.75 SD, range = 2–4, n = 6), while most early breeding season biparental broods contained four eggs and/or young (Ammon & Gilbert, 1999).

### 3.5 Post‐molt singing

Post‐molt singing was singing by territorial males which I heard when the males had completed prebasic molt. Since all males sighted in post‐molt singing were in fresh basic plumage, I considered the singing to be a likely indicator that prebasic molt was complete. I indicate the earliest and latest dates that I heard post‐molt singing (Table 1). I did not confirm the number of days an individual color‐banded male continued post‐molt singing, but for some it appeared to last about a week. The length of time males remained on breeding territories following post‐molt singing apparently was brief, and the maximum time I recorded was 4 days. Post‐molt singing was similar in pattern to singing heard earlier in the breeding season, but usually was subdued in amplitude. However, in 2001, a year when there appeared to have been an elevated late breeding season food supply in my study area, as suggested by, among other things, an unusually high number of mixed species flocks sighted (WMG, personal data), the amplitude of post‐molt singing in some Wilson's Warbler males sounded similar to that heard earlier in the breeding season.

## 4 DISCUSSION

Findings of this study indicate that many, and possibly all, resident male Wilson's Warblers in my study population remained on their breeding territories and underwent definitive prebasic molt there following their nesting season. However, the majority of resident females apparently vacated my study site following their nesting season, and presumably underwent prebasic molt elsewhere. Since I color banded 40 females over the course of the study, I likely would have sighted some of those females late in the breeding season had any been present. A small number or females did remain at my study site on or after July 21, and most, and possibly all, cared for very late broods, usually of reduced size. I never saw a female in prebasic molt, nor in fresh basic plumage, at my study site. This was further indication that most females had vacated my study site while still in worn plumage, and those that remained and raised late broods apparently delayed their prebasic molt.

Since prebasic molt in most passerine species has been found to occur at similar times for males and females (Butler et al., 2008; Flockhart, 2010; Heise & Rimmer, 2000; Vega Rivera et al., 1998), it seems possible that prebasic molt in most of the females nesting at my study site occurred, not at times greatly different from prebasic molt in males, but at sites that were different, and thus at sites other than their breeding grounds. The likely exceptions, in terms of timing of molt, were females that remained on their breeding territories and uniparentally tended late broods. Those females obviously did not molt at similar times as the males, since I sighted a male having completed prebasic molt as early as August 13, but sighted a female still in worn plumage as late as August 29. I have no information on when or where presumed prebasic molt did occur in females that remained on their territories in late‐season brood care. However, that molt likely occurred after their late broods were independent, or nearly so. This, in turn, meant that timing of prebasic molt in late‐brood‐tending females likely was very late, and likely later than prebasic molt in most, if not all, males. This presumed very late molting in late‐brood‐tending females, and their presumed subsequent late fall migration and late arrival on wintering grounds, may reflect a facultative, adaptive trade‐off between current reproductive productivity and future fitness (Hemborg, 1999; Svensson & Nilsson, 1997). The presumed very late molting in late brood‐tending females also suggests a likely extreme dichotomy in timing of prebasic molt between late‐brood‐tending females, and females that vacated their breeding territories relatively early, and possibly underwent prebasic molt soon afterwards during molt migration. Projecting forward, this would suggest that Wilson's Warblers could migrate in two waves. The first wave might be males and females that molted at similar relatively early times, and the second smaller wave might be females that uniparentally tended late broods, and molted much later.

No other study, to my knowledge, has evaluated the differential timing and location of prebasic molt between the sexes of adult Wilson's Warblers. However, some studies have provided information on mean autumn migratory passage times of Wilson's Warblers. Based on mist‐netting operations, Otahal (1995), Yong et al. (1998), and Benson et al. (2006) found no significant differences between mean autumn migratory passage times of the sexes of adult Wilson's Warblers, while (Carlisle et al., 2005) determined that adult males preceded adult females by a mean 5 days. Migratory passage times in autumn can indirectly reflect earlier events on breeding grounds (Benson & Winker, 2001). These earlier events might include molting times and strategies. Additionally, the advantages of completing molt as early as possible to facilitate earlier migration and establishment of winter territories are recognized (Rappole et al., 1979). Selection pressures therefore should logically favor molting and migration times which were as rapid as possible, although it is recognized that factors contributing to migratory timing can be complex, and can confound the premise that migration should be as rapid as possible (Carlisle et al., 2005; Otahal, 1995). Even so, three studies have determined that mean autumn migratory passage times of the Wilson's Warbler sexes are similar, and a fourth study has shown a difference of just 5 days. These findings would support a hypothesis that molt times of most individuals of the Wilson's Warbler sexes are similar, even while the locations of prebasic molt are different, as indicated by this study. Regarding the late‐nesting, uniparental females observed in this study, however, the timing and location of their prebasic molt, and the timing of their migration, are completely unknown, although both molt and migration are likely very late.

Wiegardt et al. (2017) provided evidence that a portion of Wilson's Warblers from their study sites in northern California and southern Oregon traveled upslope following breeding, and underwent prebasic molt at higher elevations. However, they also found that many individuals remained and molted on their breeding territories over the entire range of breeding elevations, and thus did not engage in molt migration. The study of Wiegardt, Barton, et al. (2017) was based on analysis of large datasets from mist net captures, and did not separate information for males and females. Wiegardt, Barton, et al. (2017) state that their results suggest a variation in strategies for molting and molt migration among Wilson's Warblers. This study may provide a more specific explanation for the dichotomy of their results. The portion of Wilson's Warblers documented as molting on their breeding territories may have been males, while the portion documented as participating in upslope molt migration may have been females.

I documented post‐molt singing occurring in most of the territorial male Wilson's Warblers that I observed following their prebasic molt. Additionally, Steele and McCormick (1995) observed Orange‐crowned Warblers (*Leiothlypis celata*) molting and/or in fresh basic plumage in mountain meadows at elevations above their breeding sites, and coined the term “molting grounds” for these high‐elevation molting sites. Although not mentioned in their 1995 note, Steele and McCormick also observed, and I confirmed, post‐molt singing in Orange‐crowned Warblers at a molting grounds (unpubl. data). Finally, Metcalf (2003–2004) documented post‐molt singing in at least five passerine species, the Warbling Vireo (*Vireo gilvus*), the American Robin (*Turdus migratorius*), the Pine Warbler (*Setophaga pinus*), the Red‐winged Blackbird (*Agelaius phoeniceus*), and the Baltimore Oreole (*Icterus galbula*). Post‐molt singing, as I here describe it for Wilson's Warblers, may be widespread in passerines and perhaps other taxa, and my observations suggest that it may be a reliable indicator that prebasic molt is complete. It should be noted, however, that post‐molt singing in passerines may not be the same as singing over extended times during non‐breeding seasons, which is reported for multiple passerine species, such as *Zonotrichia* sparrows (Chilton et al., 2020) and Townsend's Solitaires (Bowen, 2020). Post‐molt singing, as I found it occurring in Wilson's Warblers, appears to be brief, usually lasting no more than a week. Such brief singing may be found in numerous other species that otherwise are not known to sing during the non‐breeding season.

Regarding a possible adaptive function of post‐molt singing, Metcalf (2003–2004) suggested it might just be a hormonal response to changes in photoperiod. Also, it might be presumed to have a territorial function, with territorial males signaling a warning to males prospecting for future territories. This function might apply to species whose males molt on their breeding grounds, as did male Wilson's Warblers at my study site. However, since post‐molt singing in Orange‐crowned Warblers occurs on molting grounds, and based on my observations usually not on breeding grounds (unpubl. data), other adaptive functions must apply for some species.

The distinction between post‐molt singing, as I describe it for Wilson's Warblers, and extended singing during the non‐breeding season, as occurs in some other avian species, is not altogether clear, and merits further study. Similarly, the adaptive functions of singing after the breeding season, whether brief or extended, also would be of interest.

I conclude with an observation that the approach of this study involved comprehensive “boots on the ground,” field observation, and it seems unlikely that the study's relevant findings could have been obtained by another approach. This suggests that much remains to be learned about birds based on comprehensive field observation.

## CONFLICT OF INTEREST

None declared.

## AUTHOR CONTRIBUTION

**William M. Gilbert:** Conceptualization (lead); Data curation (lead); Formal analysis (lead); Funding acquisition (lead); Investigation (lead); Methodology (lead); Project administration (lead); Resources (lead); Software (lead); Supervision (lead); Validation (lead); Visualization (lead); Writing – original draft (lead); Writing – review & editing (lead).

## Data Availability

Cumulative count data for different sexes, molts, and plumage stages are accessible on Dryad (https://doi.org/10.5061/dryad547d7wm9X).
